# Impacts of climate change on tropical cyclones and induced storm surges in the Pearl River Delta region using pseudo-global-warming method

**DOI:** 10.1038/s41598-020-58824-8

**Published:** 2020-02-06

**Authors:** Jilong Chen, Ziqian Wang, Chi-Yung Tam, Ngar-Cheung Lau, Dick-Shum Dickson Lau, Hing-Yim Mok

**Affiliations:** 1 Earth System Science Programme, The Chinese University of Hong Kong, Hong Kong, China; 2 School of Atmospheric Sciences, and Guangdong Province Key Laboratory for Climate Change and Natural Disaster Studies, Sun Yat-sen University, Zhuhai, China; 3 Southern Marine Science and Engineering Guangdong Laboratory (Zhuhai), Zhuhai, China; 4 Institute of Environment, Energy and Sustainability, The Chinese University of Hong Kong, Hong Kong, China; 5 Department of Geography Resource Management, The Chinese University of Hong Kong, Hong Kong, China; 6 Hong Kong Observatory, Hong Kong, China

**Keywords:** Climate change, Atmospheric dynamics

## Abstract

We have investigated changes of western North Pacific land-falling tropical cyclone (TC) characteristics due to warmer climate conditions, using the pseudo-global-warming (PGW) technique. Historical simulations of three intense TCs making landfall in Pearl River Delta (PRD) were first conducted using the Weather Research and Forecasting (WRF) model. The same cases were then re-simulated by superimposing near- (2015–2039) and far- (2075–2099) future temperature and humidity changes onto the background climate; these changes were derived from the Coupled Model Intercomparison Project phase 5 (CMIP5) multi-model projections according to the Representative Concentration Pathway (RCP) 8.5 scenario. Peak intensities of TCs (maximum surface wind in their lifetimes) are expected to increase by ~ (3) 10% in the (near) far future. Further experiments indicate that surface warming alone acts to intensify TCs by enhancing sea surface heat flux, while warmer atmosphere acts in the opposite way by increasing the stability. In the far future, associated storm surges are also estimated to increase by about 8.5%, computed by the Sea, Lake, and Overland Surges from Hurricanes (SLOSH) model. Combined with sea level rise and estimated land vertical displacement, TC-induced storm tide affecting PRD will increase by ~1 m in the future 2075–2099 period.

## Introduction

Producing almost 30% of global tropical cyclones (TCs), the western North Pacific (WNP) is the most active TC basin on the Earth. Associated with strong winds, heavy rainfall and high storm surges, WNP TCs pose great threats to lives and cause significant economic and societal losses to cities along the Asian coastline. Adjacent to the South China Sea, the Pearl River Delta (PRD) region is one of the highly-urbanized megacity groups in China, which is also strongly affected by these storm systems^1–3^. PRD has experienced devastating floods induced by TCs related heavy rainfall and storm surges. Super Typhoon *Hato* (2017) caused a maximum storm surge of 2.79 m in Zhuhai (https://www.weather.gov.hk/informtc/hato17/hato.htm), and super Typhoon *Mangkhut* (2018) induced surges reaching 3.40 m in Hong Kong (https://www.weather.gov.hk/informtc/mangkhut18/mangkhut.htm). According to projections using general circulation models (GCMs), TCs may intensify globally^4–7^ and also sea level rise (SLR) may accelerate^8,9^ under global warming. Their combined effects will lead to increase of flood risks to the PRD area.

However, TC physics cannot be realistically represented by GCMs, due to their relatively coarse resolution^10^. Studies have also shown that there can be substantial variations in the response of TCs to climate change over different ocean basins^6,11^, while GCMs may introduce large biases when reproducing TCs activities over different regions as the result of their low resolution. By embedding high-resolution regional models within a GCM, dynamical downscaling provides a powerful way to reproduce TCs with more realistic circulation structure, intensity and frequency. For example, by further downscaling outputs from the Geophysical Fluid Dynamics Laboratory (GFDL) High Resolution Atmospheric Model (HiRAM; 50-km grid) with the GFDL Hurricane model at 6-km resolution^12^, it was found that more realistic TCs with stronger intensity can be simulated. The downscaled outputs from GFDL Hurricane model projected an overall decreased TC frequency, but with more intense storms over WNP in response to anthropogenic global warming. It was also demonstrated that there would be a continuing poleward migration of WNP TCs following the emissions projections of the Representative Concentration Pathway (RCP) 8.5, based on dynamical downscaling of 9 Coupled Model Intercomparison Project Phase 5 (CMIP5) models^13^.

Besides carrying out downscaling of outputs from individual GCMs, the impacts of warmer climate on TCs can also be explored using the pseudo-global-warming (PGW) method^14,15^. PGW is a more effective and economical way compared to traditional regional downscaling methods, and it provides a possible way to investigate responses of historical TCs to future climate change. In the PGW technique, differences of environmental thermal and dynamical forcings, such as sea surface temperature (SST), atmospheric temperature, humidity and wind field, between current and future climates simulated by GCMs are firstly obtained. These perturbations will then be added to the initial and boundary conditions for the historical cases to generate future climate conditions^15^. The PGW method has been used to examine the responses of snow storm^16^ and Baiu rain-band^17^ to future global warming, and it has become increasingly popular for investigating changes in TCs for both idealized^18^ and real cases^19–21^. Coupled with the Finite Volume Community Ocean Model (FVCOM), changes of WNP TC *Haiyan* (2013) and its induced storm surge according to four scenarios proposed by the Fifth Assessment Report (AR5) of Intergovernmental Panel on Climate Change (IPCC) were analyzed using PGW^22^. It was found that the drop of minimum sea level pressure (SLP) for TC *Haiyan* could be as large as 21 hPa, with a 2.7 m increase of maximum storm surge under RCP 8.5 if only SST warming was considered. On the other hand, such strong intensification would be significantly reduced by incorporating the effect of air temperature warming. In fact, if SST, air temperature and humidity are fully taken into account, the minimum SLP of TC *Haiyan* will just decrease by 13 hPa and the storm surge will increase by 0.7 m. Overall, the PGW technique is a useful tool for accessing how global warming might impact on weather systems, provided that storms with similar tracks appear in the future under similar synoptic conditions.

In this study, we evaluate the impacts of future global warming on WNP TCs using PGW based on the Weather Research and Forecasting (WRF) model^23^ with a horizontal resolution of 5 km, and quantify the influences on storm surges with a numerical storm surge model, namely the Sea, Lake, and Overland Surges from Hurricanes (SLOSH) model^24^. Three intense TCs that brought high storm surges in Hong Kong coastal areas are chosen and their potential responses to global warming through PGW method are investigated. The tracks, intensities and storm size information are then applied to drive the SLOSH model, in order to evaluate changes of induced storm surges in the future.

## Results

**Figure 1:**
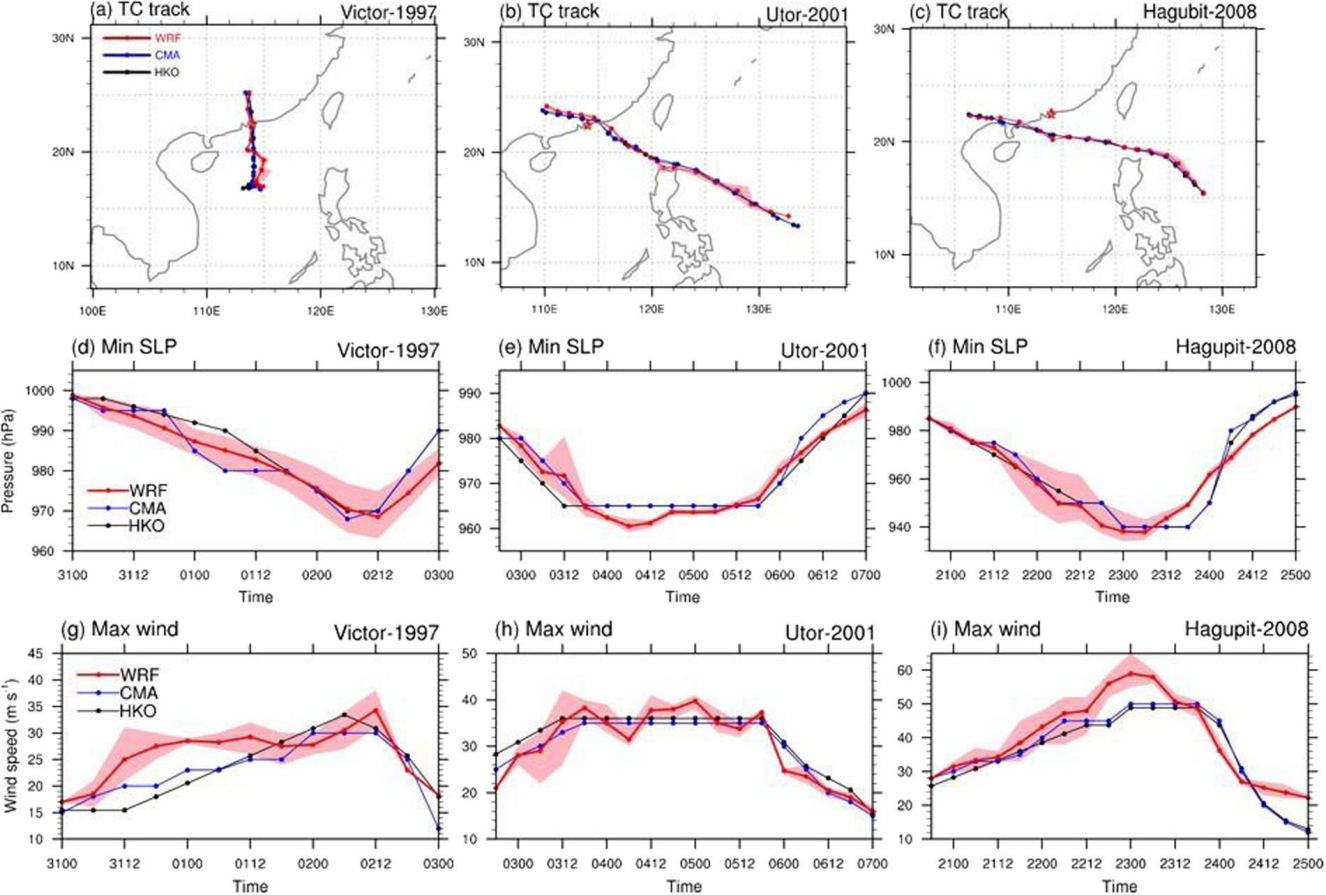
(**a**–**c**) TC tracks, (**d–f**) minimum sea level pressure and (**g–i**) maximum surface wind speed from the WRF control experiments (red curve) and TC best track data from CMA (blue curve and HKO (black curve), for (**a,d,g**) TC *Victor*, (**b,e,h**) TC *Utor* and (**c,f,i**) TC *Hagubit*. Pink shading indicates the spread among four ensemble runs with different initial conditions. The figure and maps were plotted with NCL version 6.5.0 (http://www.ncl.ucar.edu/).

First, we show that tracks and intensities of the selected TC cases can be well replicated in the WRF control experiments. As shown in Fig. 1, WRF can reasonably reproduce the characteristics of TCs. In particular, TC tracks are well captured except for TC *Victor* during its first 30 hours of integration (Fig. 1a). TC minimum SLP evolutions are also well reproduced (Fig. 1d–f), with the largest bias only 4 hPa lower than observations for TC *Utor* (Fig. 1e). On the other hand, the replication of TC maximum surface wind speed is not as good as those for tracks and minimum SLP (Fig. 1g–i). The evolution of TC *Utor* maximum wind speed is well simulated, as shown in Fig. 1h; the strength of TC *Victor* is also captured, albeit with a time evolution deviates from the observed. For TC *Hagupit*, model simulations give a peak maximum wind 8 m/s (~15%) stronger than the observed peak intensity. Based on the above, we conclude that WRF is able to replicate the three historical TCs by providing well-simulated trajectories, minimum SLP evolutions. Although the simulated evolution of maximum wind speed is not as good as the other two indices (track and minimum SLP), it is still within the ~15% bias compared to observations. Note that the differences between simulated results and observed data do not directly affect our subsequent analyses, as the response of TCs to changes in the future environments is inferred based on the relative change between the control and PGW experiments.

**Figure 2:**
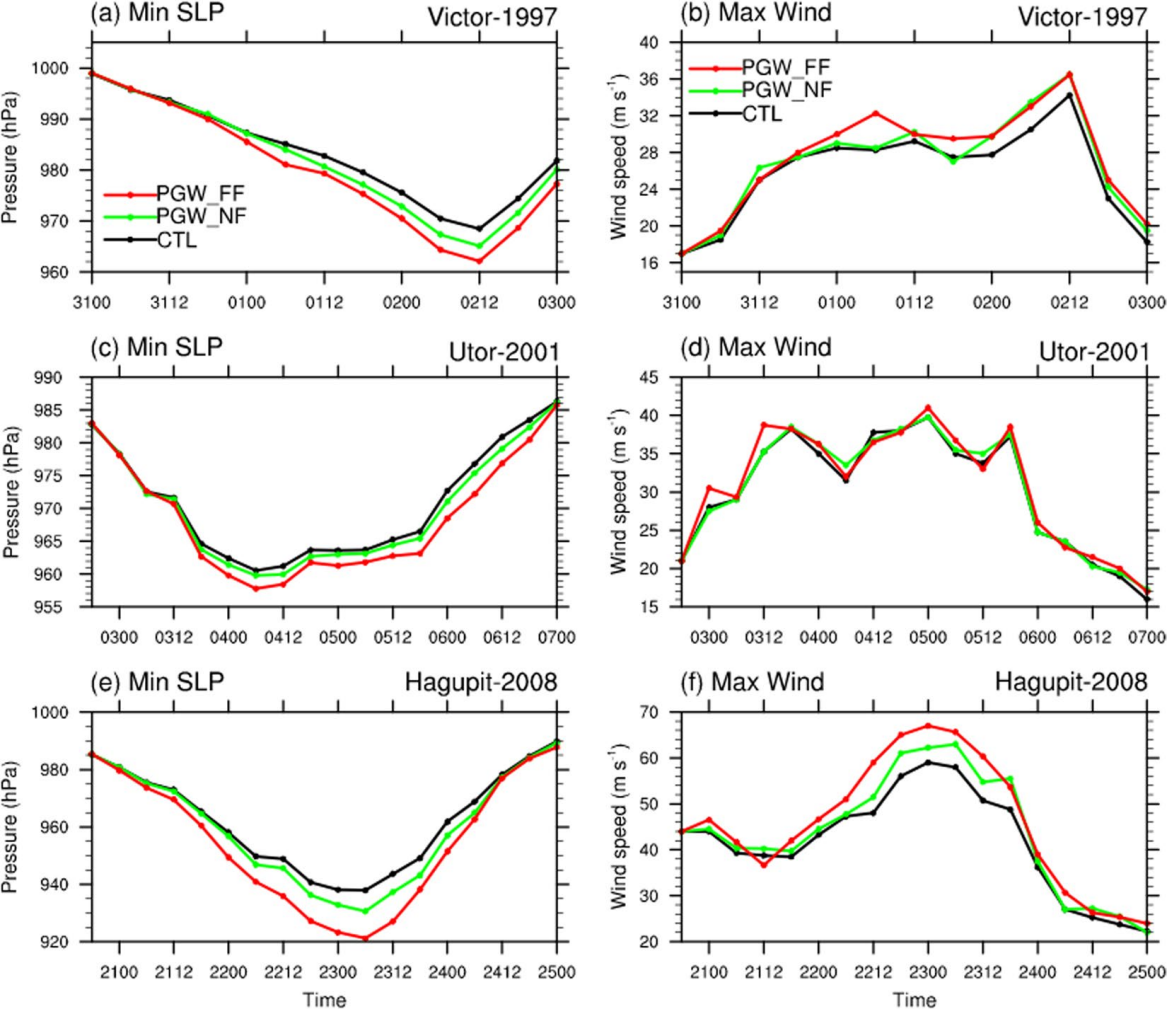
Ensemble mean (**a,c,e**) minimum sea level pressure and (**b,d,f**) maximum 10-m wind speed from the WRF control experiments (CTL, black curve) and sensitivity experiments for near (PGW_NF, green curve) and far future (PGW_F, red curve) climate changes based on the PGW method, for (**a,b**) TC *Victor*, (**c,d**) TC *Utor*, and (**e,f**) TC *Hagupit*. The figure was plotted with NCL version 6.5.0 (http://www.ncl.ucar.edu/).

We now present results based on PGW experiments for each of the TCs. Here two future periods, namely near future (2015–2039) and far future (2075–2099), are considered and for which the PGW technique is applied; in each case, ensemble integration is carried out with four ensemble members (generated using different initial times). The ensemble mean responses of TC intensity to PGW thermal effects are shown in Fig. 2. It can be seen that both in near future and far future, intensities of all three TCs are enhanced to different extents, and the intensifications are obviously stronger in the far future. For example, the minimum SLP in TC *Hagupit* is 7.2 hPa lower during the peak intensity time in the near future, while in the far future the same measure can be 16.9 hPa lower (Fig. 2e). Also, based on the peak maximum surface wind speed, the same TC will be 3.7 m/s (10 m/s) stronger in the near (far) future (Fig. 2f). On the other hand, it is also worth mentioning that different TCs respond differently to the superimposed climate change signals. For example, the response of TC *Utor* in the PGW experiments is not as sensitive as TC *Hagupit* to global warming (see Fig. 2c,d).

**Figure 3:**
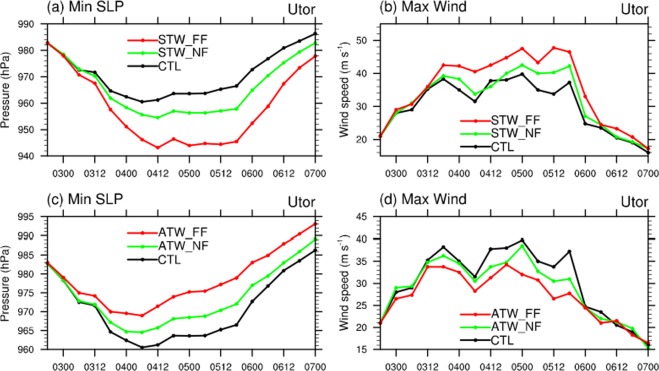
Ensemble mean (**a,c**) minimum sea level pressure and (**b,d**) maximum 10-m wind speed for TC *Utor* from the WRF control experiments (black curve) and the sensitivity experiments for the near (green curve) and far future (red curve), with climate change component due to (**a,b**) surface temperature warming (STW) and (**c,d**) atmospheric temperature warming (ATW) imposed in the PGW experiments. See text for details. The figure was plotted with NCL version 6.5.0 (http://www.ncl.ucar.edu/).

In order to further understand how the added warming signals affect TC intensity, a series of single-factor experiments are conducted. In these sensitivity experiments, warming signals are divided into surface and atmospheric parts. It should be noted that the surface part includes both land temperature rise and SST warming, though the SST warming plays the dominant role in influencing TCs. When the single-factor experiments are carried out, anomalies due to one warming factor (such as surface warming) are added, while the other factor (atmospheric warming) is set to zero. Figure 3 shows the ensemble mean results of the individual surface warming and atmospheric warming effects on TC intensity. For TC *Utor*, the added surface warming greatly enhances its intensity, with a drop of almost 20 hPa in minimum SLP during the peak intensity time, and 9 m/s stronger of maximum wind speed in the far future (Fig. 3a,b). Such a large intensification is driven by more heat flux supplied from a warmer ocean in future (see Supplementary Fig. S1). This result agrees with previous studies that demonstrate the important role of warm SST on TC intensification^25,26^. On the other hand, the atmospheric warming has a large negative impact on TC intensity (Fig. 3c,d), but the negative effect is weaker than the positive one due to SST warming. Many studies have also pointed out that atmospheric warming itself can weaken TCs, due to the associated increase of atmospheric stability^6,27^. Additionally, although the specific humidity is increased obviously in near and far futures under RCP 8.5 scenario (see Supplementary Fig. S3d), the relative humidity shows no significant change with the air warming in future^28^. As a result, the signal-factor experiments with the slight anomalies of relative humidity in WRF model present no obvious changes on TC’s intensity (figure not shown). Overall, warmer SST provides more surface heat flux to TCs over the ocean, while atmospheric warming tends to suppress TC-related convection. Nonetheless, their combined effect still leads to stronger TCs in future climate. Similar results are confirmed in the other two TC cases.

**Figure 4:**
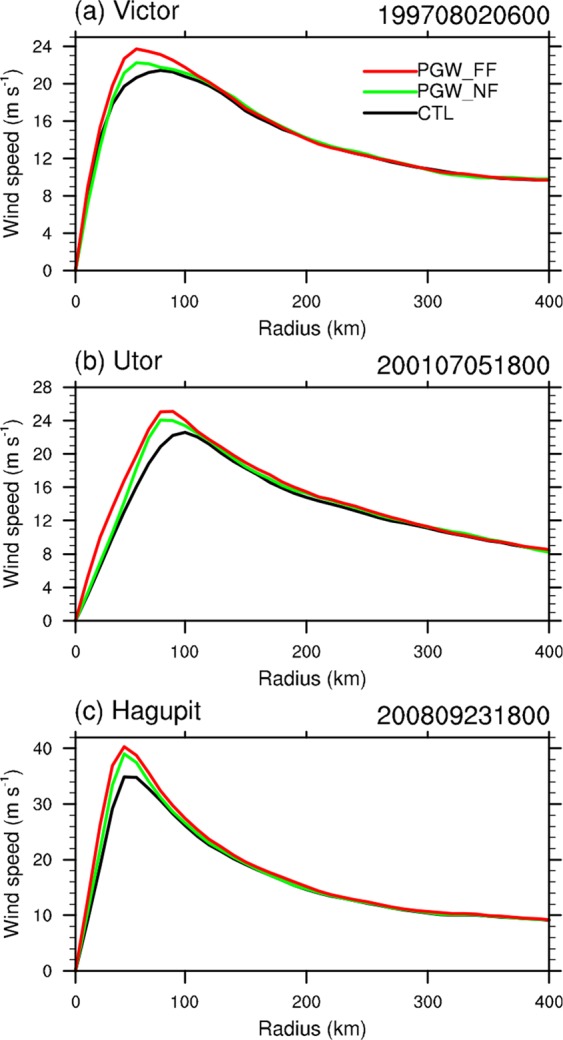
Ensemble mean azimuthal average of 10-m tangential wind from the WRF control experiments (black line) and the near (green line) and far future (red line) PGW experiments at the time before TC landfall for (**a**) TC *Victor*, (**b**) TC *Utor*, and (**c**) TC *Hagupit*. The figure was plotted with NCL version 6.5.0 (http://www.ncl.ucar.edu/).

The amplitude of storm surge is also sensitive to the radius of maximum wind speed (RMW)^29^. The response of TC RMW to PGW is also evaluated and the results are shown in Fig. 4. RMW is defined as the distance of the maximum tangential wind from TC center. RMW for all the three TCs before landfall are projected to decrease slightly in both near future and far future. The moderately decrease of RMW in response to the climate warming is not fully understood and deserves further investigation. This can also be discerned based on the azimuthally averaged TC vertical structure (represented by the tangential wind and vertical vorticity). Moreover, TC convection will be deeper and more compact in the warming future (see Fig. S2 of Supplementary Information).

**Figure 5:**
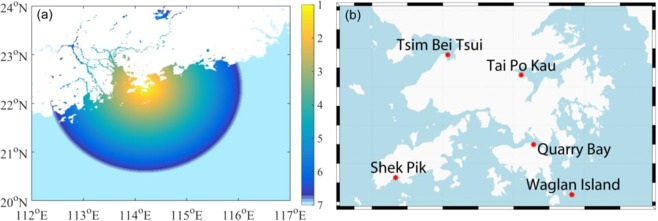
(**a**) Shading area is the model domain and grid mesh for the storm surge model SLOSH. (**b**) Locations of the five tide stations within Hong Kong Waters considered in this study. The figure and maps were plotted with MATLAB R2016a (https://www.mathworks.com/products/matlab.html).

The simulated track, intensity and RMW in both historical and PGW experiments can be used to drive the SLOSH model and evaluate the storm surge change in a warmer climate. Storm surge magnitude is influenced by a number of factors, such as TC intensity, size, storm forward speed and angle of approaching to the coast^30^. An accurate storm surge simulation requires a high quality WRF replication of all these factors for the three TCs. The shape of the local coastline can also affect the height of storm surge^30^. All the combined factors make it challenging to accurately re-simulate the storm surges. Storm surges induced by the three selected TCs were observed by five tide stations in Hong Kong, whose names and locations can be seen in Fig. 5b. It can be seen that three of the five stations are in the narrow bays and surrounded by mountainous areas, making it hard to parameterize the wind field well near the stations and to replicate storm surges. Storm surge induced by TC *Victor* (1997) is overestimated for all stations. This obvious overestimation is highly related with the simulated TC track bias. By taking close investigation of TC *Victor*’s location when approaching to Hong Kong, it can be found the observed closest distance of TC *Victor*’s center to Hong Kong is 10 km in the west. The RMW of TC *Victor* before landfall was ~70 km (See Fig. 4b), which indicates most of the tide stations were in the TC’s calm center (Hong Kong is just ~55 km from the west part to east). However, the model simulated closest distance between the TC center and Hong Kong is ~40 km, in the west of Hong Kong. Thus, all the tide stations are located in the places of close to RMW and experience much stronger winds than observed. Interestingly, Tsim Bei Tsui station is in the northeast quarter of the simulated TC when it approached to Hong Kong, experiencing the strongest wind and highest storm surge overestimation (52 cm higher). While Quarry Bay is in the east part of the TC, and is affected by the second strongest wind, introducing the second overestimation (30 cm). At the meantime, Waglan Island is in the southeast quarter of the TC, where the weakest wind exists compared with the other 3 stations, and it experiences the weakest overestimation (17 cm). Storm surge induced by TC *Hagupit* (2008) is systematically slightly underestimated (see *S/O* ratio in Table 1, where S is short for simulated and O for observed storm surge). By contrast, storm surges induced by TC *Utor* (2001) are largely underestimated, especially at Waglan Island and Shek Pik (with the *S/O* ratio less than 15%). One reason for the obvious underestimation could be that TC *Utor* (2001) has an unusually large size. It was reported by both Hong Kong Observatory (HKO) and Regional Specialized Meteorological Center (RSMC) Tokyo that the observed radius of *Utor* could reach up to more than 500 km^31,32^. However, the radius of 30 kt simulated by WRF was just within 300 km (See Fig. 4b), meaning that TC *Utor*’s whole wind structure was not adequately represented. Also, it is noted that TC *Utor* (2001) is a special case in which it made landfall north of Hong Kong; usually this kind of systems produce weaker wind because of the sheltering effect of mountains north of Hong Kong^30^. For the other two TC cases, they passed by Hong Kong to its south to southwest, which is more typical for systems causing large storm surges. In the failure of storm surge simulation for TC Utor, this case was not used in the storm surge projection. Disregarding TC *Utor*, the station-averaged simulated storm surge height for the other two TCs is 1.28 m, which is very close to the observed value of 1.25 m, with a root mean square error ($RMSE=\sqrt{\frac{1}{N-1}\mathop{\sum }\limits_{I=1}^{N}{({S}_{S}-{S}_{o})}^{2}}$, in which ${S}_{S}$ is simulated storm surge and ${S}_{o}$ is observed storm surge) of 0.44 m. Although the RMSE is a little larger than that given by HKO (0.31 m) which was obtained by comparing the observed storm surge height with SLOSH simulation of 99 TC cases^1^, considering the small sample size in this study, the simulated storm surge by SLOSH system forced by WRF outputs is still encouraging. Moreover, it should be noted that, this systematical bias may not affect our storm surge projection much, for the reason that the projected storm surge induced by TCs in the future experiments will also experience the bias in the same way. And the storm surge bias in the two TCs can be mostly removed by calculating the relative change between historical and future projection runs, because the tracks in the historical and warming runs are very similar.

**Table 1:** Observed (O) and simulated (S) storm surge (peak magnitude) at different tide stations.

TC name	Station	Observed (m)	Simulated (m)	S/O
Victor (1997)	Tsim Bei Tsui	1.13	1.65	146.0%
	Quarry Bay	1.01	1.31	129.7%
	Waglan Island	0.93	1.07	115.1%
Utor (2001)	Tai Po Kau	1.35	0.43	31.9%
	Quarry Bay	1.12	0.3	26.8%
	Waglan Island	1.16	0.15	12.9%
	Tsim Bei Tsui	1.07	0.58	54.2%
	Shek Pik	0.91	0.09	9.9%
Hagupit (2008)	Tai Po Kau	1.77	1.19	67.2%
	Quarry Bay	1.43	1.16	81.1%

**Figure 6:**
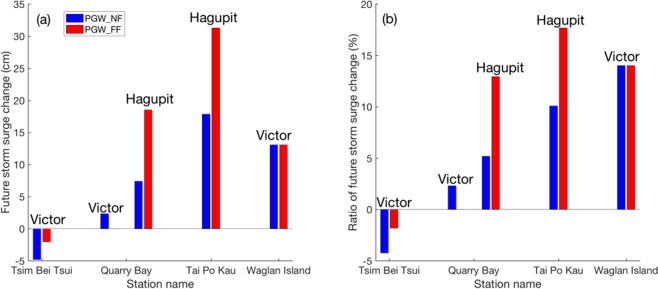
(**a**) Storm surge projected change in absolute magnitude (units: cm) and (**b**) its relative change (in %) for various TCs at the five tide stations in Hong Kong Waters. The figure was plotted with MATLAB R2016a (https://www.mathworks.com/products/matlab.html).

A systematic increase of storm surge can be seen in most of the stations in both near future and far future, except for that induced by TC *Victor* at the Tsim Bei Tsui station (see Fig. 6), which may result from the ~10 km east shift of TC track when making landfall in the near/far future, making the surface wind over the station weaker than that in historical run. On station average, storm surge amplitudes induced by the two TCs are expected to increase by 5.5% (8.6%), projected to 7.2 cm (12.1 cm) stronger in the near (far) future, compared to the observed station-averaged storm surge (1.25 m). Such a fractional increase is much weaker than its theoretical value^30^. The wind-driven surges could be simply estimated as $\zeta \propto ({\tau }_{s}/gh)W$, where $\zeta$ is the storm surge height, *g* is the gravitational acceleration, h is the depth of water, W is the shelf width and ${\tau }_{s}$ is wind stress at the air-sea interface. $\tau ={C}_{d}{\rho }_{a}{\varpi }^{2}$, in which *C*_*d*_ is the drag coefficient, ${\rho }_{a}$ is the air density and $\varpi$ is the wind speed. The TC landfall intensity is projected to increase by 7.3% in the near future and 13.9% in the far future (see Fig. 2). Thus in theory, such intensification of wind speed should enhance the storm surge magnitude by about 15% and 30% in the near and far futures, respectively.

The SLR change in Hong Kong and adjacent region is projected to rise by 65 cm in 2081–2100 relative to the period of 1986–2005 based on the multiple model simulations in CMIP5 under the RCP 8.5 scenario, and the relative SLR is expected to increase by 84 cm near Hong Kong waters if the vertical land displacement effect is also taken into consideration^33^. Combined with the enhanced TC induced storm surge, the total storm tide could be around 92 cm (74%) higher in the far future near Hong Kong waters and the neighboring PRD area on station average. However, note that the increase could be more severe for some cases; for example, storm surge induced by *Hagupit* (2008) could be 10% stronger in near future and even 17.7% stronger in the far future at Tai Po Kau station, implying 18 cm (31 cm) higher in the near (far) future, compared to the observed storm surge 1.77 m induced by TC *Hagupit* at Tai Po Kau station. Combined with far future SLR, the induced total storm tide could be almost 108 cm higher in the 2075–2099 period due to global warming, assuming the astronomical tide will not change.

## Discussion

In this study, we investigate the responses of three WNP TCs to global warming thermal effects through the PGW technique. It is found that the three TCs will be intensified in both near future (2015–2039) and far future (2075–2099). The minimum SLP will be reduced by 3.6 (7.9) hPa, while the maximum surface wind speed is expected to increase by 3% (10.2%) in the near (far) future. Such intensification is basically consistent with previous studies^5,12^. To further understand the physical process of global warming affecting to TC intensity in the future periods, a series of single-factor experiments are conducted by adding surface (land and ocean) warming and atmospheric warming individually. It is concluded that surface warming can greatly intensify TCs by providing much more sea surface heat flux, while future atmospheric condition offsets the intensification effect by stabilizing the atmosphere. Additionally, TCs’ RMW will be reduced in the warming future. By incorporating the WRF simulation results with SLOSH model, impacts of global warming on the storm surge could be evaluated. On average, storm surge will increase systematically by 5.5% (8.5%) in the near future (far future). Combined with the SLR effect based on the RCP 8.5 scenario, storm tide could be 92 cm higher on average in the 2075–2099 period in Hong Kong waters and the neighbouring PRD region. It is worth pointing out that the value could be stronger for strong TCs and at some stations; for instance, storm tide induced by TC *Hagupit* at Tai Po Kau could be 108 cm higher in far future.

PGW allows researchers to examine the change of characteristics of historical weather systems under global warming conditions. If a similar TC system appears in the future under a warmer climate, it can become more destructive in the area. On the other hand, here we do not consider the impacts of recurrent modes of climate variability on TCs affecting the region. The genesis and intensification shifted by El Niño-Southern Oscillation (ENSO), for instance, as indicated by previous studies^34,35^ are not addressed here. More work needs to be done to examine how changes in the storm frequency and tracks over the WNP due to global warming might affect the overall statistics of TC wind and induced storm surge in the PRD region.

## Methods

### WRF model settings and data description

In order to investigate the effects of global warming on WNP TCs and their induced storm surges, 3 intense historical TCs that invaded to PRD region from different directions and brought high storm surges to the PRD region are chosen for simulations. TC *Victor* in 1997 (Fig. 1a) was generated in South China Sea and made landfall directly at south part of Hong Kong with a 60 Knots landfall intensity and brought the maximum storm surge 1.13 m at the Tsim Bei Tsui station (Fig. 5b). Typhoon *Utor* (2001; see Fig. 1b) hit northeast part of Hong Kong with a peak intensity of 70 Knots and induced a 1.35 m storm surge at the Tai Po Kau station (see Fig. 5b). As shown in Fig. 1c, Typhoon *Hagupit* (2008) had a peak intensity of 95 Knots and passed southwest of Hong Kong bringing storm surge as high as 1.77 m at Tai Po Kau station. The storm surge data can be found in https://www.hko.gov.hk/wservice/tsheet/pms/stormsurgedb_e.htm.

The model used here to simulate the three TCs is Advanced Research WRF (WRF-ARW) dynamical core (version 3.7)23, with a single domain of 5-km horizontal resolution and 45 vertical layers (and model top at 30 hPa). Such high spatial resolution is applied in order to better capture TC circulation including the minimum SLP and the structure of the eyewall^10,36^. The size and coverage of the model domains are shown in Fig. 1a–c, varying from case to case, determined by the trajectory of each cyclone. The physical packages used in this study include the WRF Single-Moment 6-Class (WSM6) microphysics scheme, the Kain-Fritsch convective scheme, the Noah land surface model, the Yonsei University planetary boundary layer scheme, the Dudhia shortwave radiation, and the Rapid Radiative Transfer Model for longwave radiation (more details of the model physics description could be seen at http://www2.mmm.ucar.edu/wrf/users/phys_references.html). The initial and boundary conditions for the runs are obtained from ERA-Interim reanalysis data produced by European Centre for Medium-Range Weather Forecasts (ECMWF)^37^, whose spatial resolution is approximately 80 km (T255 spectral) with 60 levels in vertical. The boundary conditions (including SST) are updated on every 6 hours. Additionally, the best track data^32^ including observed TC track, size, minimum SLP, and maximum surface wind speed from both HKO and China Meteorological Administration (CMA) are used here to evaluate the performance of WRF simulations on TC.

Simulation for TC *Victor* began on July 31 at 00:00 UTC and the model was integrated for 78 hours. For TC *Utor*, the run was initialized on July 2 2001 at 12:00 UTC and it was integrated for 108 hours. Integration for TC *Haigupit* started on September 20 2009 at 12:00 UTC and was carried out for 108 hours. The initial times are chosen to leave sufficient time for TC spinup (0.5–1 day) and to provide appropriate initial conditions that reproduce realistic trajectories and structures. To ensure that TC tracks in the model are reasonably close to observations, model zonal and meridional winds above 500 hPa are spectrally nudged towards ERA-Interim reanalysis values with a wavelength larger than 1000 km, so as to provide a realistic steering flow in the model but not to touch the inner core circulations of the simulated TCs. In order to obtain a more realistic and accurate TC intensity and location in the initial condition, Rankine vortices structures are inserted to the initial data based on the real TC intensity and location through the WRF bogus scheme. Additionally, in order to simulate more reasonable weather conditions and TC circulation, each TC case was simulated with 4 slightly different initial conditions (each with 6 hours apart) and the ensemble mean of the output data were analyzed.

### SLOSH model

Storm surge induced by the three TCs can be numerically simulated by the SLOSH model. SLOSH is developed by the National Weather Service (NWS) of the National Oceanic and Atmospheric Administration (NOAA), and is widely used for storm surge flooding forecast and risk assessment in the US^24,29^. The model equations are based on the shallow water system^29^. For the wind stress parameterization, *C*_*D*_, the surface drag coefficient, is empirically defined as a constant 3 × 10^−6^ (multiplied by the ratio of air/water density). The wind friction coefficients *k*_*s*_ and *k*_*n*_, in tangential and radial direction, are artificially defined as ${k}_{s}=1.15{k}_{n}=\alpha {[\frac{{10}^{-4}R}{0.3{V}_{R}+60}]}^{1/2}$in SLOSH^29^, where *R* is the radius of maximum wind speed, *V*_*R*_ is storm’s maximum wind speed, and $\alpha$ is a constant. It can be inferred that stronger storm wind speed can reduce the wind fraction coefficient and lead to weaker convergence, to avoid an overestimation of storm surge in strong surface winds. To estimate the heights of storm surge resulting from a TC, SLOSH requires TC track, atmospheric pressure and storm size as input parameters to generate the wind field using a simplified parametric wind model. In this paper, the three selected 6-hourly TC trajectories, minimum SLP and radius of maximum wind (RMW) simulated by WRF are adopted by SLOSH. And these six-hourly inputs are then interpolated into hourly data for driving the model. The storm surge output for each location is in 10-minute time steps. On the other hand, the existing hyperbolic SLOSH domain of Hong Kong is centering at Hong Kong and covers the whole PRD region outreach the basin 200 km far away. It was created by NOAA, and has been using since the start of the operation in 1990s. Since SLSOH does not take into account the rainfall amount, river flow or wind-driven wave, therefore wind wave generated by the storm including low period wave are not included in the SLOSH. Also, significant storm surge usually occurs when the TC is close to the coast, and the storm surge is minimal when the TC is far away. Therefore, a large basin may not be required. But the minimum size of domain should be 2.5 times of the storm size suggested by NOAA. Since storm size is 56 km in general, and a 200-km domain of Hong Kong is large enough. SLOSH starts to compute the storm surge at least 48 hours before a TC approaching Hong Kong and up to 24 hours after the time of closest approach. The resolution is 7 km in horizontal in the open sea and gradually increased to 1 km near the coastline (see Fig. 5a).

### Pseudo global warming technique

In the control run, the three chosen TC cases are reproduced with WRF, and the responses of these TCs to global warming are then evaluated by applying the PGW method. We firstly get the information of future climate change, namely the differences of monthly mean variables (surface temperature, atmospheric temperature, and relative humidity) between present (1975–1999) and the near/far (2015–2039/2075–2099 in RCP 8.5 scenario) future climates based on the CMIP5 multi-model ensemble mean (totally 31 models listed in Table S1 of Supplementary Information). The obtained anomalies for each variable are then added to the 6-hourly ERA-Interim initial and boundary conditions for the three TC cases to build new conditions in the pseudo-warming environment. The changes of the three factors in July for the near and far futures according to the RCP 8.5 scenario are displayed in Supplementary Fig. S3. SST will be around 3 °C warmer (Fig. S3b) in the far future. And the near surface temperature will be ~3 °C warmer, with stronger warming in the upper level (near 250 hPa), leading to the atmosphere being more stable (Fig. S3c) in future. The specific humidity will also increase by 4 g/kg near the surface level (Fig. S3d) in the same above period. Note that the variable of relative humidity is used in the WRF model, and the relative humidity transferred from the specific humidity in troposphere shows no significant change in the warming future^28^ (figure not shown). The three TCs are re-simulated with the newly built initial and boundary conditions in both near future and far future by using the same WRF settings as in the control runs. By comparing these near future and far future results with the control runs, we can therefore infer the responses of TCs to future global warming. It should be noted that we do not add changes of the wind field in the PGW simulations due to following reasons: (1) the wind field does not show significant change over WNP in the warming future; (2) different GCMs predict different trend of circulation in WNP, which is unlike SST, atmospheric temperature and specific humidity (all models predict an increasing trends for all these 3 factors); the system may not be in balance if the anomalous wind field is added; (3) to estimate the future change of induced storm surges using PGW, storm systems similar to the three TCs are assumed to occur in the future; changes in winds are not considered so as to avoid obvious changes of storm tracks.

### Projecting storm surge changes from model to observation

It could be seen from Table 1 that there are biases in the SLOSH simulations when compared to the observed storm surge heights, due to the TC biases from positions, intensities and radii of maximum winds. It might not be appropriate to use the projected change of storm surge magnitude by SLOSH directly. Here, the projected change of the storm surge in SLOSH is converted using the projected relation S_p_ = S_obs_ × (S_pgw_ − S_his_)/S_his_, where S_p_ is the projected change of storm surge in the future and S_obs_ is observed historical storm surge, while S_pgw_ and S_his_ are SLOSH simulated future and historical storm surge changes.

SLR may reduce the magnitude of TC-induced storm surge. Based on the formula $\zeta \propto ({\tau }_{s}/gh)W$, the storm surge ${\rm{\zeta }}$ is in inverse relation with the water depth h, SLR can increase water depth and reduce storm surge magnitude. The average water depth around Hong Kong waters is around 20 m^38^. The relative SLR in Hong Kong waters is 0.84 m, thus the relative reduced storm surge can be simply estimated by $\frac{{\rm{\delta }}{\rm{\zeta }}}{{\rm{\zeta }}}=\frac{{\rm{SLP}}}{{\rm{h}}}\approx 4 \%$. The storm surge can be reduced ~4% in the far future when combining the SLR effect.

## Electronic supplementary material

Supplementary information.
