# Supplementary material for: Impacts of climate change on tropical cyclones and induced storm surges in the Pearl River Delta region using pseudo-global-warming method
Source: Sci Rep. 2020 Feb 6;10:1965. doi: 10.1038/s41598-020-58824-8 (PMC7005268; doi:10.1038/s41598-020-58824-8)
Supplement: Supplementary file 1 — Supplementary information. [file 41598_2020_58824_MOESM1_ESM.pdf]

Supplementary Information

Table S1. Information about the 31 CMIP5 models used in this study.

Model name	Modeling group
ACCESS1.0	Commonwealth Scientific and Industrial Research Organization and Bureau of Meteorology (CSIRO-BOM)
ACCESS1.3	Same as above
BCC-CSM1.1	Beijing Climate Center (BCC), China Meteorological Administration
BNU-ESM	College of Global Change and Earth System Science (GCESS), Beijing Normal University (BNU)
CanESM2	Canadian Centre for Climate Modelling and Analysis(CCCMA)
CCSM4	National Center for Atmospheric Research (NCAR)
CESM1-BGC	Same as above
CESM1-CAM5	Same as above
CMCC-CM	Centro Euro-Mediterraneo sui Cambiamenti Climatici (CMCC)
CNRM-CM5	Centre National de Recherches Météorologiques (CNRM)/Centre Européen de Recherche et Formation Avancée en Calcul Scientifique (CERFACS)
CSIRO-Mk3-6-0	CSIRO in collaboration with Queensland Climate Change Centre of Excellence (CSIRO-QCCCE)
FGOALS-g2	State Key Laboratory of Numerical Modeling for Atmospheric Sciences and Geophysical Fluid Dynamics (LASG), Institute of Atmospheric Physics, Chinese Academy of Sciences
FGOALS-s2	Same as above
GFDL-CM3	National Oceanic and Atmospheric Administration (NOAA) Geophysical Fluid Dynamics Laboratory (GFDL)
GFDL-ESM2G	Same as above
GISS-E2-H	National Aeronautics and Space Administration (NASA) Goddard Institute for Space Studies (GISS)
GISS-E2-R	Same as above
HadGEM2-AO	National Institute of Meteorological Research/KoreaMeteorological Administration (NIMR/KMA)
HadGEM2-ES	Same as above
INM-CM4	Institute for Numerical Mathematics (INM)
IPSL-CM5A-	L'Institut Pierre-Simon Laplace (IPSL)

LR	
IPSL-CM5A-MR	Same as above
IPSL-CM5B-LR	Same as above
MIROC-ESM	Japan Agency for Marine-Earth Science and Technology (JAMSTEC), Atmosphere and Ocean Research Institute (AORI; University of Tokyo), and National Institute for Environmental Studies (NIES) AORI (University of Tokyo), NIES, and JAMSTEC
MIROC-ESM-CHEM	Same as above
MIROC5	Same as above
MPI-ESM-LR	Max Planck Institute for Meteorology (MPI-M)
MPI-ESM-MR	Same as above
MRI-CGCM3	Meteorological Research Institute (MRI)
NorESM1-M	Norwegian Climate Centre (NCC)
NorESM1-ME	Same as above

Figure S1. Turbulent heat flux change in near and far future

To ascertain how SST warming might influence TC intensity, future change of daily mean turbulent heat flux one day before TC *Utor* making landfall, based on the PGW experiments, is computed and shown in Fig. S1. Enhanced latent heat flux is seen over South China Sea in both near future (Figs. S2a and b) and far future (Figs. S1c and d), consistent with the stronger TC intensity in these experiments.

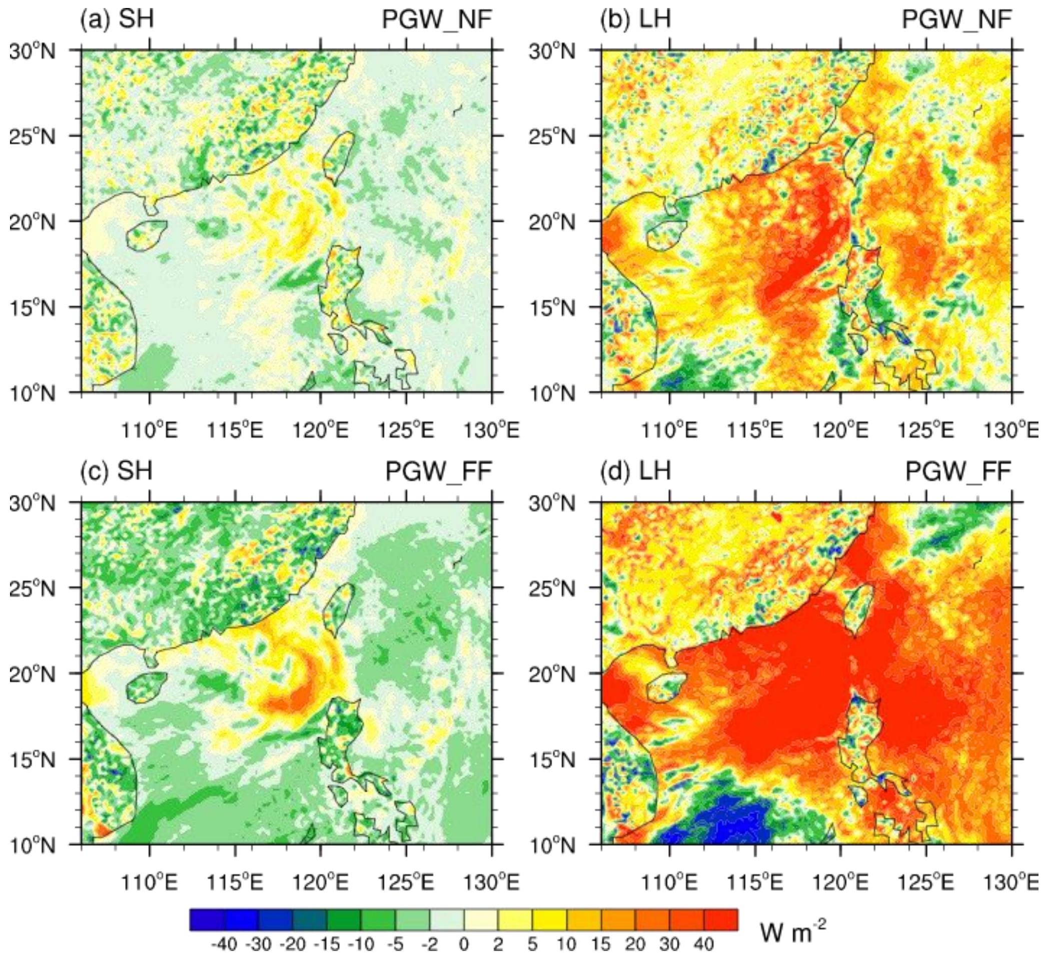

Figure S1. Ensemble mean differences of daily mean (a, c) sensible heat flux and (b, d) latent heat between (a, b) near future, (c, d) far future PGW experiments and the control experiment, for TC *Utor* one day before its landfall. The figure and maps were plotted with NCL version 6.5.0 (http://www.ncl.ucar.edu/).

Figure S2. Change in TC vertical structure in response to global warming

Fig. S2 shows the relative vorticity and tangential wind for TC *Utor* from different experiments. It can be seen that the TC related vorticity is enhanced and extending deeper near its center, but weaker in outer region of the eye in both near future (Fig. S2d) and far future (Fig. S2e). This implies the TC becomes more compact and extends more in the vertical under a warmer climate.

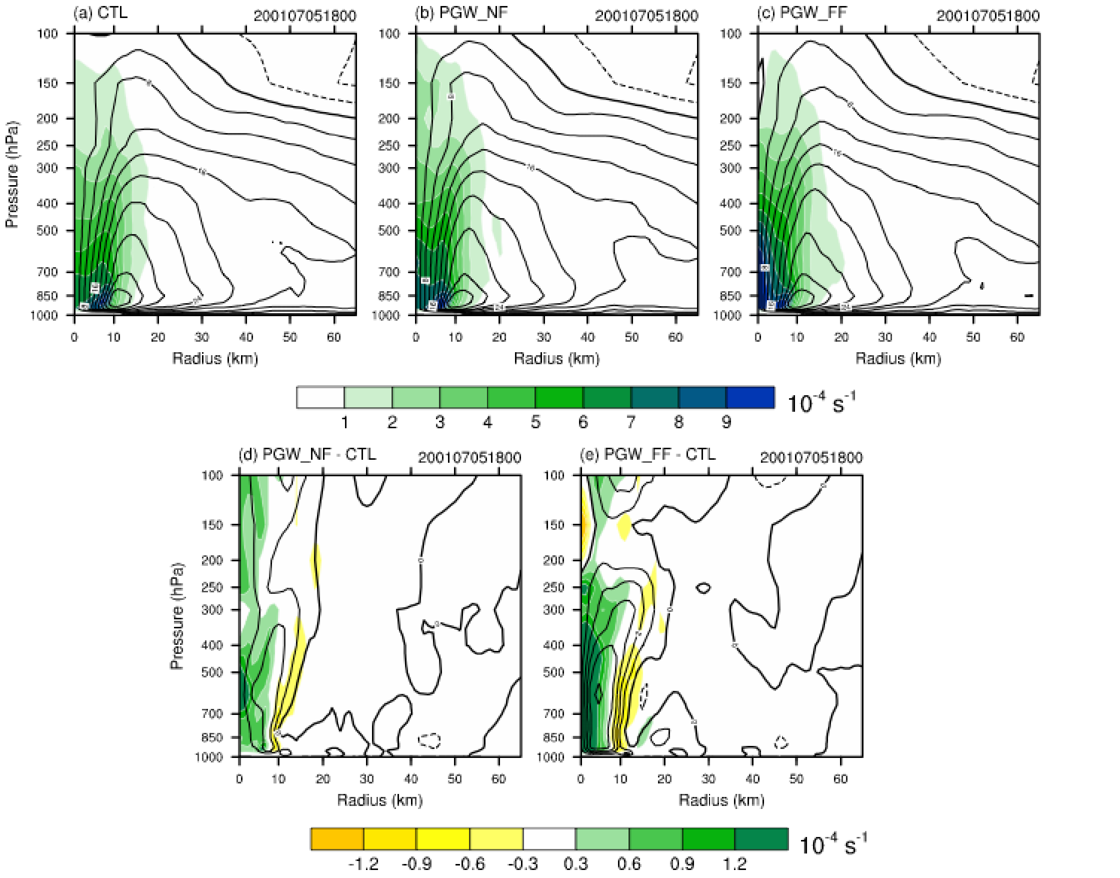

Figure S2 Ensemble mean azimuthally averaged tangential wind (counter lines) and vorticity (shading) from (a) control experiment, (b) the near future and (c) far future PGW experiments at 1800 UTC 5 July 2001 (before landfall) for TC *Utor*. (d) Difference between PGW_NF and CTL. (e) Difference between PGW_FF and CTL. The figure was plotted with NCL version 6.5.0 (http://www.ncl.ucar.edu/).

Figure S3. Changes in the background state in near and far future

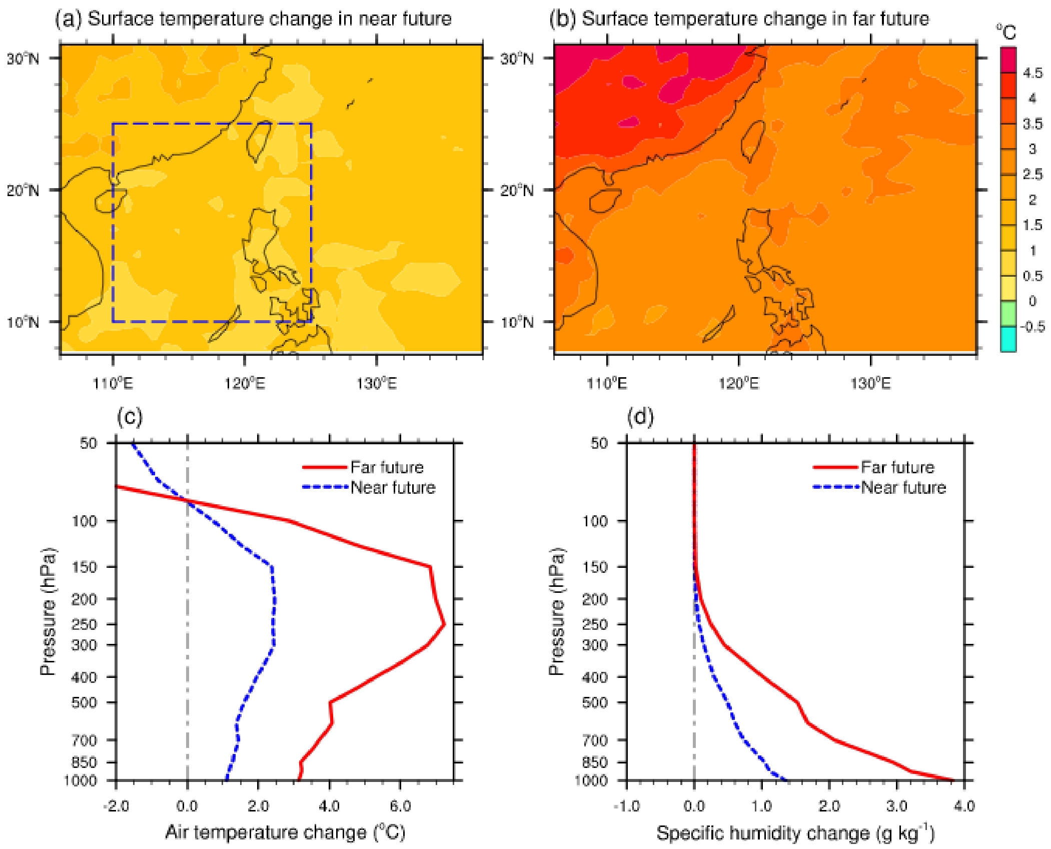

Figure S3. (a) Surface temperature change in near future (2015-2039) and (b) far future (2075-2099) in July from CMIP5 multi-model mean according to the RCP 8.5 scenario, relative the historical period (1975-1999). Near-future and far-future change in area-averaged (c) air temperature and (d) specific humidity, over the domain of 10-25°N, 110-125°E. The figure and maps were plotted with NCL version 6.5.0 (http://www.ncl.ucar.edu/).
